# A Common *BACE1* Polymorphism Is a Risk Factor for Sporadic Creutzfeldt-Jakob Disease

**DOI:** 10.1371/journal.pone.0043926

**Published:** 2012-08-30

**Authors:** Olga Calero, María J. Bullido, Jordi Clarimón, Ana Frank-García, Pablo Martínez-Martín, Alberto Lleó, María Jesús Rey, Isabel Sastre, Alberto Rábano, Jesús de Pedro-Cuesta, Isidro Ferrer, Miguel Calero

**Affiliations:** 1 Centro de Investigación Biomédica en Red sobre Enfermedades Neurodegenerativas (CIBERNED), Madrid, Spain; 2 Unidad de Encefalopatías Espongiformes, Centro Nacional de Microbiología, Instituto de Salud Carlos III (CNM-ISCIII), Madrid, Spain; 3 Centro de Biología Molecular Severo Ochoa (CSIC-UAM), Madrid, Spain; 4 Institute of Sanitary Research “Hospital la Paz” (IdIPaz), Madrid, Spain; 5 Neurology Department, Hospital de la Santa Creu i Sant Pau, Universitat Autònoma de Barcelona, Barcelona, Spain; 6 Neurology Service, Hospital Universitario La Paz (UAM), Madrid, Spain; 7 Alzheimer Disease Research Unit, CIEN Foundation, Carlos III Institute of Health, Alzheimer Center Reina Sofia Foundation, Madrid, Spain; 8 Banco de Tejidos Neurológicos Universidad de Barcelona-Hospital Clínico, Barcelona, Spain; 9 Banco de Tejidos de la Fundación CIEN, CIEN Foundation, Carlos III Institute of Health, Alzheimer Center Reina Sofia Foundation, Madrid, Spain; 10 Área de Epidemiologia Aplicada, Centro Nacional de Epidemiología, Instituto de Salud Carlos III (CNM-ISCIII), Madrid, Spain; 11 Institute of Neuropathology (INP), IDIBELL-Hospital Universitari de Bellvitge, Faculty of Medicine, University of Barcelona, Hospitalet de LLobregat, Barcelona, Spain; “Mario Negri” Institute for Pharmacological Research, Italy

## Abstract

The β site APP cleaving enzyme 1 (BACE1) is the rate-limiting β-secretase enzyme in the amyloidogenic processing of APP and Aβ formation, and therefore it has a prominent role in Alzheimer’s disease (AD) pathology. Recent evidence suggests that the prion protein (PrP) interacts directly with BACE1 regulating its β-secretase activity. Moreover, PrP has been proposed as the cellular receptor involved in the impairment of synaptic plasticity and toxicity caused by Aβ oligomers. Provided that common pathophysiologic mechanisms are shared by Alzheimer’s and Creutzfeldt-Jakob (CJD) diseases, we investigated for the first time to the best of our knowledge a possible association of a common synonymous *BACE1* polymorphism (rs638405) with sporadic CJD (sCJD). Our results indicate that *BACE1* C-allele is associated with an increased risk for developing sCJD, mainly in *PRNP* M129M homozygous subjects with early onset. These results extend the very short list of genes (other than *PRNP*) involved in the development of human prion diseases; and support the notion that similar to AD, in sCJD several *loci* may contribute with modest overall effects to disease risk. These findings underscore the interplay in both pathologies of APP, Aβ oligomers, ApoE, PrP and BACE1, and suggest that aging and perhaps vascular risk factors may modulate disease pathologies in part through these key players.

## Introduction

Alzheimer’s (AD) and Creutzfeldt-Jakob (CJD) diseases are distinct fatal neurodegenerative pathologies sharing common epidemiologic and pathophysiologic mechanisms [1], including the deposition of disease specific pathognomonic amyloidogenic proteins [1]–[5], pathways involved in amyloid generation and clearance [1], [5], [6], and genetic and vascular risk factors [3], [7], [8]. Recently, we have shown a genetic cross-interaction between *APOE* and *PRNP,* the major genetic risk factors [9]–[11] in the sporadic forms of AD and CJD [12].

In AD, the amyloid deposits consist predominantly of a peptide of 39–43 amino acids called amyloid-β peptide (Aβ) [13], [14], resulting from the pathogenic processing of the amyloid β precursor protein (APP) by sequential cleavage by β-secretase and γ-secretase [15], [16]. In CJD, when the deposition occurs, the amyloid consists of a protease-resistant and aggregated fragment derived from the cellular prion protein (PrP) isoform [17]–[21].

β-secretase, also called β site APP cleaving enzyme 1 (BACE1), is a membrane-bound aspartyl protease involved as the rate-limiting enzyme in the amyloidogenic processing of APP and Aβ formation [22]. An increased BACE1 activity that correlates with Aβ peptides load has been observed in the brain of sporadic AD patients [23]–[25]. Recent evidence suggests that PrP protein interacts directly with BACE1 regulating its β-secretase activity [25]–[27]. Griffiths *et al* suggested [25] that the interaction of PrP with the N-terminal prodomain (residues 22–45) of the immature, Golgi-localized form of BACE1 results in a retention of BACE1 in the secretory pathway and a reduced presence at the cell surface and endosomes where it should exert its secretase function on APP.

Some authors have reported the association between *BACE1* polymorphisms and the risk of developing AD, although this link is still matter of discussion. However, at present there are no association studies of *BACE1* polymorphisms with prion diseases. In this paper, we investigated for the first time a possible association of a common synonymous *BACE1* polymorphism at codon 262 (NM_012104.3:c.786G>C, rs638405) with sporadic CJD (sCJD).

## Results

**Figure 1:**
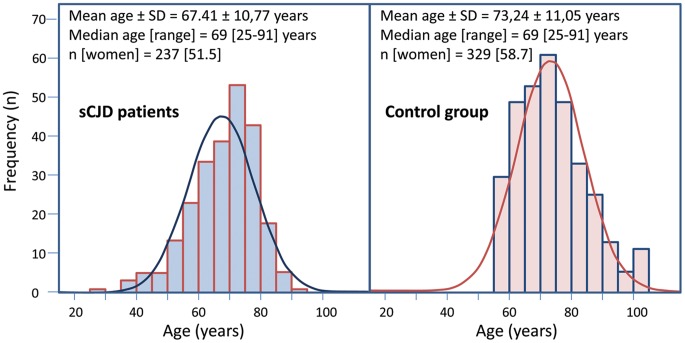
Demographic description and histogram of age distribution at clinical onset for sCJD (left) and sample procurement for controls (right) adjusted to normal distribution curves. Statistically significant differences between sCJD and control populations were observed for age (p<0.001) but not for gender distribution (p = 0.14).

We have analyzed the risk of sCJD associated with a *BACE1* polymorphism (rs638405) and their potential interaction with *PRNP*. For this purpose, the study population included 237 sCJD patients and 329 healthy controls. The demographic description of these populations is shown in Figure 1.

Age distributions for the populations analyzed followed non-normal distributions. We found statistically significant age differences between sCJD and control populations (p<0.001). However, no statistically significant differences were observed in gender distribution (p = 0.14). No statistically significant differences were found between definitive and probable sCJD cases for any of the parameters studied.

The allelic and genotypic distributions of *BACE1* and *PRNP* genes were in Hardy-Weinberg equilibrium in the populations studied, with the exception of *PRNP* in sCJD. We observed an increased risk of sCJD for individuals homozygous at codon 129 in the *PRNP* gene (OR = 5.01, CI 95% = 3.29–7.62, p = 5.5×10^−14^) as previously reported for a similar population [12] and in agreement with previous reports worldwide. The distributions of *BACE1* genotypes stratified by age and the polymorphic codon 129 of *PRNP* are shown in Table 1.

**Table 1:** *BACE1* (rs638405) and *PRNP* codon 129 genotypic frequencies in control subjects and sCJD patients.

	*BACE1*	n (%)	Age at onset(years)		129 *PRNP* genotypic frequency, n (%)		
	Genotypes		<71	≥ 71	M129M	M129V	V129V
**Control subjects**	CC	108	41	67	38	52	18
		(32.8)	(28.7)	(36.0)	(29.9)	(32.1)	(45.0)
	CG	145	68	77	57	73	15
		(44.1)	(47.5)	(41.4)	(44.9)	(45.1)	(37.5)
	GG	76	34	42	32	37	7
		(23.1)	(23.8)	(22.6)	(25.2)	(22.8)	(17.5)
	Total	329	143	186	127	162	40
		(100.0)	(100)	(100)	(100.0)	(100.0)	(100.0)
**sCJD cases**	CC	89	54	35	55	19	15
		(37.6)	(40.9)	(33.3)	(35.3)	(43.2)	(40.5)
	CG	109	58	51	80	15	14
		(46.0)	(43.9)	(48.6)	(51.3)	(34.1)	(37.8)
	GG	39	20	19	21	10	8
		(16.5)	(15.2)	(18.1)	(13.5)	(22.7)	(21.6)
	Total	237	132	105	156	44	37
		(100.0)	(100)	(100)	(100.0)	(100.0)	(100.0)

The analysis of *BACE1* polymorphism effect on sCJD (Table 2) showed a non-significant association trend for *BACE1* C-allele carriers (OR = 1.56, p = 0.052) for the whole population. Post-hoc power analysis of this result, assuming that the probability of exposure (C+ carriers) is 0,769 for controls, and 0.835 for cases, indicated that we are able to reject the null hypothesis that the odds ratio equals 1 with probability (power) of 0.629 for an alpha error (type I) equal to 0.05, in a model of inequality of proportions for two independent groups by Fisher′s exact test. Analogous power analysis by using a logistic regression model and assuming a binomial distribution for the independent variable (*BACE1* polymorphism) yielded a slightly lower power value of 0.536.

**Table 2:** Odds ratios for the association between sCJD and *BACE1* C-allele carriers at rs638405 among different strata defined by *PRNP* codon 129 genotypes.

	All subjects		Onset before 71 y		Onset at or after 71 y	
129 *PRNP* genotypes	OR (95% CI)	p-value	OR (95% CI)	p-value	OR (95% CI)	p-value
All subjects	1.56 (1.00–2.43)	0.052	1.97 (1.03–3.77)	**0.039**	0.94 (0.57–1.56)	0.82
M129M	2.46 (1.29–4.69)	**0.006**	3.99 (1.51–10.5)	**0.005**	1.79 (0.74–4.34)	0.20
M129V	0.95 (0.41–2.20)	0.91	0.93 (0.33–2.68)	0.89	1.09 (0.27–4.50)	0.90
V129V	0.55 (0.16–1.89)	0.34	0.23 (0.10–5.06)	0.35	1.03 (0.21–5.11)	0.97

However, after stratification by *PRNP* genotypes, a clear association was found within the *PRNP* M129M homozygous stratum (OR = 2.46, CI 95% = 1.29–4.69, p = 0.006). Interestingly, the *BACE1* C-allele risk appears to increase with increasing numbers of *PRNP*-M129 alleles (Table 2).

As described in the methods section, this study is composed of two distinct sCJD populations coming from the Carlos III Institute of Health, Madrid, Spain (ISCIII, n = 166) or the Institute of Neuropathology (INP), IDIBELL-Hospital Universitari de Bellvitge, Barcelona, Spain (INP-IDIBELL, n = 71). In order to check for results consistency, we analysed the ISCIII sCJD cases and used the INP-IDIBELL cases as internal replication population. For both populations, we found a consistent association of *BACE1* C-allele with sCJD exclusively in the *PRNP* M129M homozygous stratum: OR = 2.16, CI 95% = 1.10–4.25, p = 0.026 for ISCIII population*;* OR = 4.82, CI 95% = 1.33–17.45, p = 0.016 for INP-IDIBELL population.

In order to explore the influence of age on this risk factor, cases and controls were divided into two groups according to the median age for these populations pooled together (onset before and onset at or after 71 years old). Analysis of *BACE1* polymorphism risk on different age strata indicated that the association of *BACE1* C-allele found in the M129M homozygous stratum was mainly driven by individuals with early onset (OR = 3.99, 95% CI = 1.51–10.49, p = 0.005) (see Table 2).

These results suggested a potential interaction between *BACE1* and *PRNP* genes. In order to measure the size and significance of the possible interaction, we performed a synergy factor (SF) analysis. Taking as reference those individuals without these traits (*PRNP* M129V heterozygous and *BACE1* GG), we observed that *PRNP* M129M homozygous subjects had a 2.46-fold increased risk of developing sCJD, while the risk associated with *BACE1* C-allele carriers was 0.94. When these genotypes were analyzed jointly, subjects carrying both traits (*PRNP* M129M homozygous and *BACE1* C-allele carriers) had a 6.09-fold increased risk of developing sCJD than subjects without these traits. Synergy analysis yielded a SF of 2.63, which was not statistically significant (p = 0.059). However, after stratification by age (cut-off at 71 years old), a SF of 4.21 (p = 0.037) was obtained for subjects with onset before 71 years old, indicating that interaction between these two genes was age dependent. Similar analysis taking as reference *PRNP* V129 carriers (M129V+V129V) and *BACE1* GG yielded a significant SF of 2.82 (p = 0.020). For individuals with early onset (<71 years old) we also observed a significant interaction with a SF of 4.29 (p = 0.022). Further analysis by a logistic regression model controlled by age, sex and *PRNP* homozygosis and using the number of *PRNP*-M129 alleles and the *BACE1*-C allele status as covariables indicated that the interaction factor “*PRNP*-M129 alleles”×“*BACE1*-C allele status” was statistically significant (p = 0.016).

Additionally, we explored the influence of *BACE1* polymorphism on disease duration and age at onset in sCJD population, but we did not observe any significant association between these factors in *BACE1* C-allele carriers compared to *BACE1* GG individuals (data not shown).

## Discussion

Here, we have analyzed for the first time the distribution of *BACE1* rs638405 polymorphism on a sCJD population. Our results indicate that *BACE1* polymorphism is associated with risk for developing sCJD, which is mainly driven by *PRNP* M129M homozygous subjects. The effect of *BACE1* polymorphism appears to be age-dependent, being more relevant in earlier onset sCJD patients, where a genetic interaction is observed between *BACE1* and the major susceptibility marker for human prion diseases (*PRNP*).

BACE1 is a type 1 transmembrane aspartic protease with a key role on Aβ formation [28], [29], and in the production of the amyloid precursor protein intracellular domain (AICD) that plays a role in transcriptional transactivation [30]. Recently, it has been suggested that the cellular form of prion protein, PrP^C^, participates in a feedback loop controlling Aβ production [31], [32]. Moreover, PrP^C^ has been proposed as the cellular receptor of toxic Aβ oligomers [33], [34]. In AD, the feedback loop appears to be disrupted by the binding of Aβ oligomers to PrP^C^, preventing BACE1 regulation [31], [32]. According to this, higher levels of BACE1 activity may modulate the production of key players of AD and prion pathologies by increasing the production of both Aβ and PrP via AICD signalling [27]; although this model has been recently questioned [35].

Since BACE1 does not participate in PrP processing [31], alterations of BACE1 activity could not directly explain the abnormal metabolism of PrP in sCJD. However, as the processing of APP by BACE1 is a key step for the production of Aβ peptides, and AICD in turn up-regulates PrP expression, increased levels of BACE1 could be related to higher cellular levels of PrP that are associated with increased susceptibility to prion diseases [36]. Moreover, other secretases, such as ADAM10, are involved both in APP α-cleavage and PrP shedding [37], [38]. In a cellular context, BACE1 and ADAM10 are in competition for their substrates [39]; and changes in the levels of one of them perturb the levels of the other [28]. Therefore, it is likely that an increase of BACE1 activity may modulate ADAM10 activity, leading to PrP expression and shedding perturbations *in vivo* [37], [40]. In this sense, increased levels of BACE1 activity have been consistently found in CSF of AD patients [41]–[48], as well as in sCJD [42]. However, opposite to AD, in sCJD increased levels of BACE1 activity in CSF did not correlate with higher levels of BACE1 protein in brain. Since amyloid plaques have been detected in sCJD brain patients [49]–[51], these results suggest a disturbed Aβ metabolism in sCJD [42], [52], [53].

As *BACE1* rs638405 is a silent polymorphism, a mechanism independent of the primary sequence of BACE1 protein may be involved in the potential functional expression of this SNP. Interestingly, we found that *BACE1* rs638405 may exert its effect by altering the activity of exonic splicing enhancers (ESE). Specific analysis of ESE regions predicted the presence of two ESE sequences in the G-allele that are abolished in the C-allele within the regions TGAT(G/C)A and ATGAT(G/C); therefore, rs638405 potentially influences *BACE1* gene expression or splicing. Alternatively, it is possible that this polymorphism serves as a tag SNP not directly involved in the pathogenic mechanism, but in linkage disequilibrium with other regulatory SNP [54], [55].

Besides *PRNP*, a small number of genes have been proposed to be associated with human prion diseases, although with inconsistent results [56]–[59]. We have recently reported that homozygosity at codon 129 of *PRNP* act synergistically as risk factor for sCJD with other genetic factors such as the *APOE* ε4 allele [12] or polymorphisms at the *CALHM1* gene [60]. Very recently, a relatively large genome-wide association study (GWAS) has suggested additional genetic risk factors in human prion disorders [61], although the necessarily small sample size and heterogeneity of the disease have probably precluded the positive identification of additional genes.

In summary, our results indicate that *BACE1* rs638405 is associated with an increased age-dependent risk for developing sCJD. Unfortunately, this study is necessarily limited by the small number of sCJD cases as it is a very rare disease. However, it must be taken into account that the sCJD population studied represents a big portion of cases diagnosed in Spain (a country with a comparatively large 45-million population), many of them with a post-mortem confirmed diagnosis. The inclusion of sCJD populations registered abroad – for instance from the EuroCJD consortium-will surely provide more universal validity to the results, but it would also carry an increased genetic variability to the data of this already heterogeneous disease that may in turn preclude the stratified analysis performed in this study.

In our view, the novelty and relevance of the present study lean on the following facts: i) it represents the first association study of *BACE1* in sCJD, ii) these findings extend the very short list of genes (other than *PRNP*) involved in the development of prion diseases in humans; and support the notion that similar to AD, in sCJD several *loci* may contribute with modest overall effects to disease risk [12], [62], iii) stratification and analysis of the interaction with other risk genes have been revealed as a potent tool to unveil hidden effects in general analyses, and it may in part explain the lack of consensus in the analysis of *BACE1* association studies in AD [62]–[64], and iv) these results, together with our recently published works [12], [60], open up an interesting research line regarding common genetics risk factors, and points to the interplay in both pathologies of APP, Aβ oligomers, ApoE, PrP and BACE1 with other key players such as aging and vascular risk factors [3], [7], [8], [65].

## Materials and Methods

### Subjects

The populations of analysis included 237 sCJD cases and 329 subjects with normal cognitive status. All subjects were Caucasians of Spanish origin. Control samples were obtained from the Neurology Departments of Hospital La Paz (Madrid) and the Hospital Clínico San Carlos (Madrid) (n = 268), and National Centre of Microbiology at the Carlos III Institute of Health, Madrid, Spain (n = 61). All samples from sCJD cases analyzed in this study were obtained from patients with suspected prion diseases, submitted for diagnostic purposes under the guidelines of the Spanish National Referral and Surveillance system analyzed at the Institute of Neuropathology (INP), IDIBELL-Hospital Universitari de Bellvitge, Faculty of Medicine, University of Barcelona, Hospitalet de LLobregat, Barcelona, Spain (n = 71) or at the National Centre of Microbiology at the Carlos III Institute of Health, Madrid, Spain (n = 166). For some sCJD cases, clinical and neuropathological information was retrospectively gathered from the Spanish National Registry of Human Transmissible Spongiform Encephalopathies. Among the 237 sCJD cases, 187 (78.9%) were definitive, neuropathologically verified patients, while 50 (21.1%) were probable sCJD cases. Genetic cases of human prion diseases were excluded after complete DNA sequencing of *PRNP* coding region.

### Ethics Statement

The study was approved by the Bioethics and Animal Welfare Committee from the Instituto de Salud Carlos III, Madrid, Spain; and by the Ethical Research Committees from Universidad Autónoma de Madrid, Madrid, Spain. Control samples were obtained with the adequate understanding and written consent of subjects. All the data were analyzed anonymously, and clinical investigations have been conducted according to the principles expressed in the Declaration of Helsinki.

### DNA Analysis

Total DNA was isolated from peripheral blood or cerebral tissue following standard procedures. Genotyping of *BACE1* polymorphism (rs638405) was determined by TaqMan® probes (assay code: C_3058171_1; _Applied Biosystems) as described by the manufacturer. This polymorphism is located at position 28149 of *BACE1* RefSeqGene NG_029372 (version NG_029372.1, GI:339409206) on human chromosome 11. The analysis of the polymorphism at codon 129 of the *PRNP* gene (rs1799990) was performed by DNA sequencing using specific primers [66]. No new data on sequence variability of the genes analyzed was found.

### Functional Prediction Analysis

Functional prediction analysis of *BACE1* rs638405 was performed by the use of *FuncPred* online software (http://snpinfo.niehs.nih.gov/snpfunc.htm) [67]. Exonic splicing enhancers were analyzed by RESCUE-ESE Web Server (http://genes.mit.edu/burgelab/rescue-ese/) [68].

### Statistical Methods

Statistical analyses of nominal or categorical variables were performed by Fisher’s exact text or Pearson’s chi-square test. Quantitative variables (age at onset, disease duration) did not follow a normal distribution and were analyzed by non-parametric statistical hypothesis contrast with Mann-Whitney U test. Unconditional logistic regression models controlled by age and gender were used to compare genotypic and allelic frequencies and to calculate association adjusted odds ratio (OR) and 95% confidence intervals (CIs). The Hardy-Weinberg test for genotype frequency distributions was performed on the observed genotype frequencies for each population, with significance based on a standard observed-expected chi-square distribution with one degree of freedom. Deviation from normality of quantitative variables was checked by the Kolmogorov-Smirnov statistic with Lilliefors’ significance. The synergy factor (SF), confidence intervals and significance were calculated as described [69], [70]. Most statistical analyses were performed with PASW Statistics 19 software. Post-host power analyses of association data were performed by either Fisher′s exact test for a model of inequality of proportions for two independent groups or z-test for a logistic regression model by using the G*Power 3.1.3 software [71] based on the implementation of the procedure of Hsieh and collaborators [72].
